# The effect of mandibular advancement on pharyngeal airway space in patients with oral squamous cell carcinoma: A monocentric prospective study with computed tomography

**DOI:** 10.1007/s00784-025-06147-1

**Published:** 2025-01-14

**Authors:** Lucas M. Ritschl, Jakob K. Zink, Tobias Unterhuber, Jochen Weitz, Benedikt Hofauer, Klaus-Dietrich Wolff, Andreas M. Fichter, Alexandra V. Behr

**Affiliations:** 1 Department of Oral and Maxillofacial Surgery, Klinikum Rechts Der Isar, School of Medicine and Health, Technische Universität München, Ismaninger Str. 22, Munich, D-81679 Germany; 2 Practice Clinic of Oral and Maxillofacial Surgery and Dentistry, Traunstein, Germany; 3 Department of Oral and Maxillofacial Surgery, Josefinum, Augsburg and Private Practice Oral and Maxillofacial Surgery Im Pferseepark, Franz-Kobinger Strasse 7a, Augsburg, 86157 Germany; 4 Department of Otorhinolaryngology, Head and Neck Surgery, Medical University of Innsbruck, Innsbruck, Austria

**Keywords:** Mandibular advancement device, Protrusion, Posterior airway space, Computer tomography, Segmentation, Obstructive sleep apnea

## Abstract

**Objectives:**

The presented study aimed to evaluate the effect of mandibular protrusion with a temporarily applied mandibular advancement device (MAD) on the posterior airway space and to determine a reliable metric constant based on a three-dimensional computed tomography (CT) evaluation.

**Materials and methods:**

The study population consisted of patients with oral squamous cell carcinoma who were treated at least six months prior to the follow-up CT in supine position. Each patient received an individually adjusted MAD that was temporarily applied with three different protrusion distances (P_0_ = 0 mm, P_4_ = 4 mm, and P_8_ = 8 mm) during follow-up CT. The open-source software Slicer was used to calculate three parameters: minimum cross-sectional area (minCSA), mean cross-sectional area (meanCSA), and volume.

**Results:**

The results showed a significant increase for all three parameters. The minCSA increased as follows: P_0_ = 236.4 mm^2^ ± 192.2; P_4_ = 309.2 mm^2^ ± 235.4; and P_8_ = 430.6 mm^2^ ± 265.3. The meanCSA increased significantly (*p* < 0.001) in all protrusion steps and all parts of the pharynx. The volume changed as follows: P_0_ = 24.0 cm^3^ ± 5.0; P_4_ = 29.6 cm^3^ ± 18.1; and P_8_ = 33.6 cm^3^ ± 19.0. The minCSA increased by 24.9 mm^2^ ± 13.0 per millimeter mandibular protrusion.

**Conclusion and clinical relevance:**

The results are interesting for both conservative and surgical therapy and could find future application in dental, orthodontic, and combined oral surgical therapy. With the results of this study, surgeons and dentists may better predict the change of PAS parameter in order to better prepare for orthognathic surgery. They also could ensure the right protrusion distance for mandibular advancement devices in the case of obstructive sleep apnea.

## Introduction

Obstructive sleep apnea (OSA) is one of three sleep-related breathing disorders, with a prevalence of 3–7% among men and 2–5% among women [1]. Clinical symptoms of OSA cover a wide range, from snoring, excessive daytime sleepiness, and headache up to heart failure and higher cardiovascular morbidity. Patients with a body mass index (BMI) > 35 kg/m^2^ have a higher risk of suffering from OSA [2, 3]. In addition to the gold standard diagnostic tool of polysomnography, 2D and 3D imaging of the posterior airway space (PAS) can help to identify and localize the problem of obstructive sleep apnea and help in therapy decision making [4]. Three-dimensional analysis of the pharyngeal airway space has become popular in the last decade. Technical improvements in hardware and software, as well as the increasing spread of cone-beam computed tomography (CBCT) in dental and oral surgical practices, have allowed surgeons to diagnose and correlate obstructive airway anatomy and to accurately plan orthognathic surgeries with the help of a 3D computer-assisted workflow. Computed tomography (CT) is considered a useful predictive tool, with significant differences in these measurements being verified between asymptomatic patients and patients with OSA [5]. Usually, patients are treated in an interdisciplinary approach by specialists in sleep medicine, specialists in otorhinolaryngology, dentists, or oral and maxillofacial surgeons. Interdisciplinary collaboration is very important because the causes of OSA are multifactorial and show both neural and pharyngeal anatomical aspects. The first and most commonly used therapy for OSA remains nasal continuous positive airway pressure (nCPAP), which is used for all severities of OSA [6]. Unfortunately, nCPAP therapy is very compliance-dependent, which is hindered by a 50% drop-out rate or use for less than four hours per night [5, 7]. Another conservative treatment option is the use of mandibular advancement devices (MAD). There has been an increase in the number of articles suggesting that the therapeutic approach to OSA with MADs has proven to be effective in improving the parameters of polysomnographic indices and objective and subjective measures of sleepiness [5, 8], especially when they are individual and titratable [9]. Cavaliere et al. suggested that MAD treatment response in their studied population was 91%, with the mean apnoea–hypopnoea index (AHI) improving from 43.10 to 12.93 [10]. It has also been reported that a combination of nCPAP and MAD requires lower pressures to be used, which can increase compliance with nCPAP therapy [11]. Drug-induced sleep endoscopy is used alongside polysomnography and CT to evaluate the effectiveness of MAD therapy. Effective treatment alternatives to refractory to conservative treatment are the implantation of a hypoglossal stimulator or orthognathic surgery addressing mandibular-maxillary advancement. Both are common procedures with positive effects on the PAS [12–15]. The aim of orthognathic surgery is the advancement and counterclockwise rotation of the mandibular in order to increase the minimum cross-sectional area, the mean cross-sectional area, and the volume of the pharyngeal airway and to protect the airway from collapsing tissue [16]. The literature describes an effective increase in pharyngeal airway space parameters after orthognathic surgery. However, the study population is often small and imaging is performed with CBCT in a standing position or only cephalometry is used [14, 17]. Even though mandibular-maxillary advancement is a reliable and effective procedure [14], the quantitative effect of mandibular protrusion on the PAS is unknown, even though fixed functional appliances showed positive effects regarding pharyngeal airway dimensions [18]. Further, Giralt-Hernando et al. described in their systematic review and meta-analysis how only a few studies performed three-dimensional and volume analysis of the pharyngeal airway space [19].

The aim of this monocentric prospective study was to evaluate the temporary effect of controlled mandibular protrusion on the PAS parameters and in this way to achieve a better correlation between the metrically adjustable sagittal mandibular advancement and the effect on the PAS parameters.

## Materials and methods

### Ethical statement

All clinical investigations were conducted according to the principles expressed in the Declaration of Helsinki. This prospective study was approved by the institutional ethics committee of the Technische Universität München, Klinikum rechts der Isar (Approval Number: 727/20S).

### Study collective

Patients were recruited from the Department of Oral and Maxillofacial Surgery, Klinikum rechts der Isar, Technische Universität München, Munich (Germany) while regularly receiving a CT scan as part of cancer follow-up as recommended in the German national guidelines [20]. For patients to be included in the study, at least six months must have passed since their intraoral reconstruction surgery due to oral squamous cell carcinoma (OSCC). Moreover, only tumor patients who were due to receive a CT as part of their regular follow-up care were included, so as not to allow healthy patients to undergo a series of three CTs. This corresponds to the ALARA principles (as low as reasonably achievable). Flap volume decreases the most within the first six months after reconstruction (after surgery alone 84.5% or surgery and radiotherapy 96% of total expected shrinkage) [21, 22]. The postoperative swelling decreases in the first three months [23] and has mostly subsided completely within 5–8 months [24]. An interval of at least six months between the operation and the first measurement was therefore chosen. In another study, it was also shown that the PAS parameters no longer change significantly after the first phase [25]. Exclusion criteria were as follows: (1) patient age less than 18 years, (2) missing antagonistic anterior teeth for sufficient attachment of the MAD, (3) limited jaw opening (< 20 mm) or (4) jaw protrusion (< 8 mm), (5) significant pharyngeal abnormities, and (6) local or systemic contraindications for a MAD. The most common forms of reconstruction in the patient cohort were the radial forearm free flap (48%), a local flap (20%), the fibula free flap, and the soleus perforator flap (both 10%). Every patient had to answer the STOP BANG questionnaire once. This is a clinically tested arrangement of questions designed to estimate the individual risk of the patient suffering from OSA [3]. As this was an exploratory cross-sectional study, no pre- and postoperative comparison could be made with the more commonly used OSAS questionnaires (SF-36 and Functional Outcomes of Sleep Questionnaire) [26]. A declaration of consent was obtained from all patients (informed and written consent).

### Mandibular advancement device and data acquisition

**Fig. 1:**
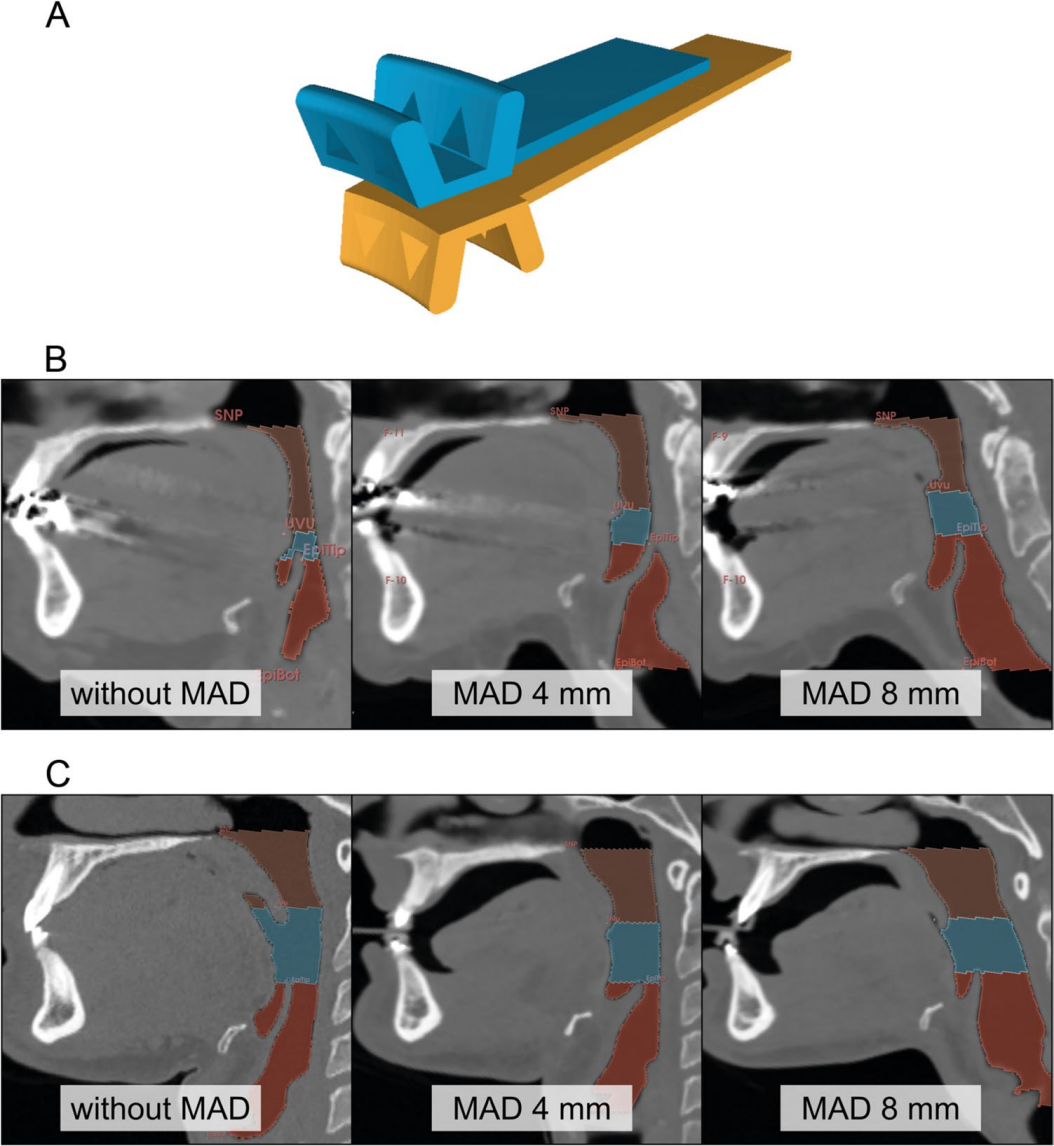
Schematic representation of the MAD (**A**) using the planning software Autodesk Fusion 360 (Autodesk Inc. 2020), as well as two cases without the MAD and a dental, temporarily fixed MAD in the different mandibular protrusion positions P_4_ and P_8_ (**B** and **C**)

A MAD was planned with the construction software Autodesk Fusion 360 (Autodesk Inc. 2020). They were designed with retention cutouts for the impression material, slim in order not to have to open the mouth widely and with a flat surface for continuous shifting against each other. The MAD contained two components (one for the maxilla and one for the mandible), which could be moved against each other separately to adjust the exact amount of mandibular advancement (0 mm = P_0_, 4 mm = P_4_, or 8 mm = P_8_) (Fig. 1). Two sizes of MAD were used: one for a wide row of teeth and one for a narrow row of teeth. These MADs were printed by a fused deposition modeling printer (Anycubic Mega X printer; Shenzhen Anycubic Technology Co. Ltd., China) using medical-grade filament (Trayfill Filament, Bernhardt Kunststoffverarbeitungs GmbH, Germany). Individual adaption of the MADs to each patient’s row of teeth was performed with the help of an alpha-silicon impression material (HS-A-Silikon fast, Henry Schein Dental Deutschland GmbH, Germany). For each CT examination one of three protrusion distances was randomly selected for the MAD, were calibrated and only worn for the duration of the CT scan. In the case of P_0_, patients underwent CT imaging in maximal intercuspation without a MAD as the control group.

During the CT scan (Philips IQon Spectral CT, Koninklijke Philips N.V., Netherlands) in the Department of Diagnostical and Interventional Radiology of Klinikum rechts der Isar, Technische Universität München, Munich, Germany) the MAD with either P_0_, P_4_, or P_8_ advancement was temporarily fixed intraorally. The CT slice thickness was 0.9 mm with a pixel spacing of 0.76 mm. Patients were instructed to keep their mouth closed, to breathe through their nose, not to swallow, and not to move. If patients were unable to do so, so that segmentation and measurement weren’t meaningful, the patients were excluded afterward. All patients were CT-scanned in supine position. This is more diagnostically conclusive regarding OSA than in vertical position [27].

### Measurements and analyses

**Fig. 2:**
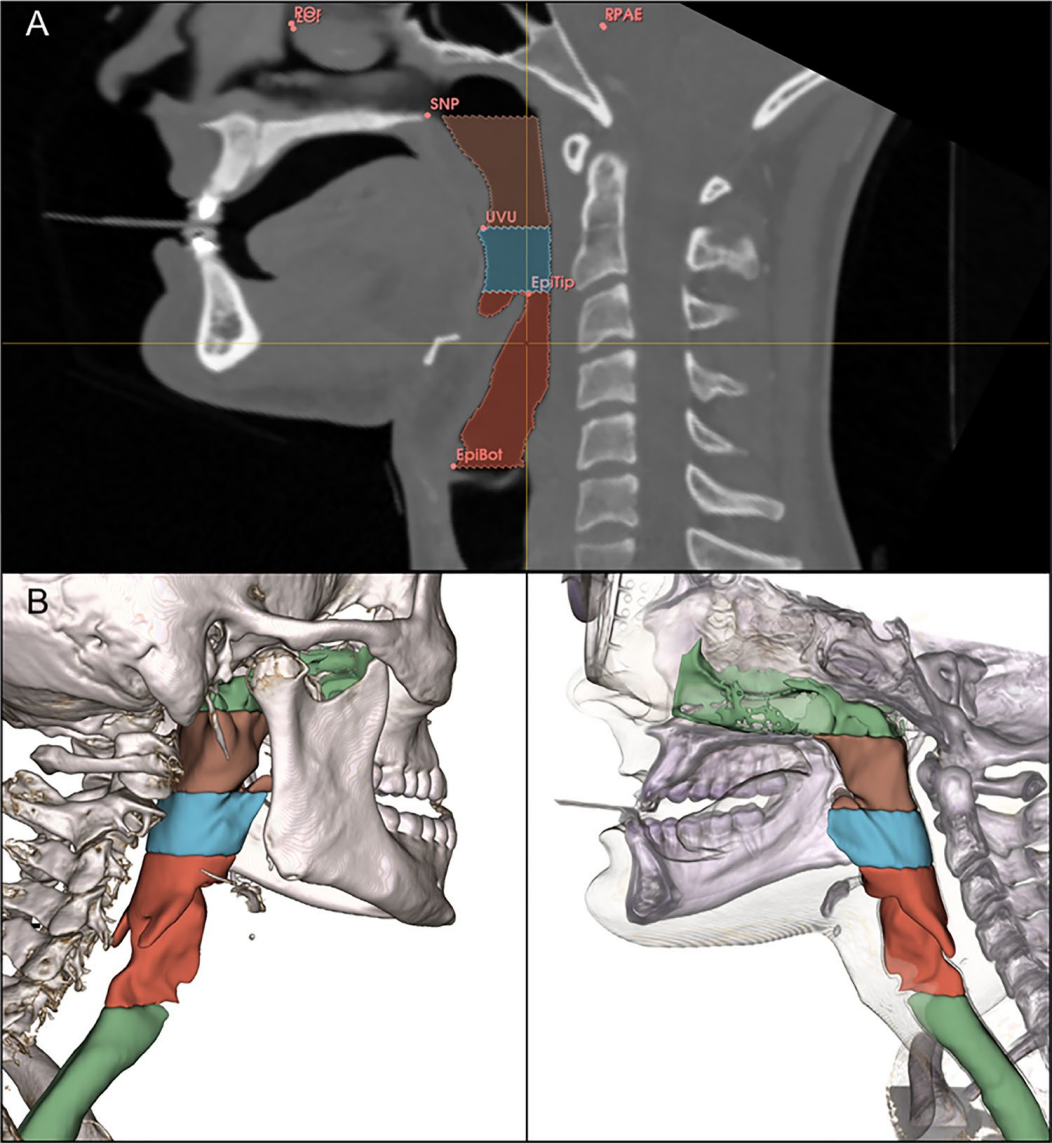
(**A**) Following anatomical landmarks represented the boundaries of the pharyngeal airway and were used for CT-scan alignment and measurements: SNP = *posterior nasal spine*; RPAE = *right porus acusticus externus*; UVU = *uvula tip*; EpiTip = *epiglottal tip*; EpiBot = *epiglottal bottom*. (**B**) Three-dimensional rendering of the pharyngeal airway without (left) and with (right) soft tissue contour

DICOM datasheets of the CT examination were imported into 3D Slicer Software version 4.13.0 [28]. 3D Slicer Software has been used in many previous medical studies with great success [29, 30]. First, various anatomical landmarks for orientation and boundary were set according to similar studies [31–35]: LOr (left orbitale), ROr (right orbitale), RPAE (right porus acusticus externus), LPAE (left porus acusticus externus), SNP (posterior nasal spine), UVU (tip of the uvula), EpiTip (tip of the epiglottis), and EpiBot (bottom of the epiglottis). Second, each patient’s DICOM set was aligned to the Frankfurt horizontal plane using LOr, ROr, LPAE, and RPAE (Fig. 2A). The examiner was blinded to the protruded distances (P_4_ or P_8_).

Third, the posterior airway was segmented with the “grow from seeds” function from 3D Slicer and adjusted manually if needed. The “grow from seeds” function is an automated segmentation algorithm using differences in the density of radiographic tissues. These different density points are manually set by the instructor. Fourth, the segmentation was divided into three regions of interest: nasopharynx (from PNS to UVU), oropharynx (from UVU to EpiTip), and hypopharynx (from EpiTip to EpiBot). Fifth, the minimum cross-sectional area (minCSA), mean cross-sectional area (meanCSA), and volume of each region were computed using “sandbox extensions” in 3D Slicer. MinCSA was calculated as the minimum cross-sectional area of the two sections naso- and oropharynx. Calculating minCSA for the hypopharynx isn’t expedient due to the motility of the epiglottis and the collapsible cartilage structure of the larynx. MeanCSA is the arithmetic mean of all cross-sectional areas in the naso, oro-, and hypopharynx.

### Statistical analysis

Given the exploratory design and character of the study, it is not necessary to calculate the sample size. Statistical analysis was performed with JASP software (JASP version 0.16.1, University of Amsterdam, Netherlands). Volume and meanCSA were calculated for each section of the airway (naso-, oro-, and hypopharynx) as well as the sum of these three for each patient for up to three different protrusion distances (P_0_, P_4_, and P_8_). All measurements were performed by one rater (JZ) and, in line with similar studies, 15 patients were randomly selected and measured again for intra-rater correlation testing [36]. An intra-class correlation coefficient (ICC) greater than 0.8 is referred to as high consistency [37]. We used a linear mixed model for ANOVA analysis and estimating fixed effects in order to get the change in the PAS parameters as a linear function model. The advantage of using a linear mixed model was that rows with missing data weren’t excluded and all observations could be used for analysis. All statistical tests were performed at an exploratory two-sided 5% significance level.

## Results

### Patient data

Fifty Caucasian patients (28 men and 22 women) were eligible for this study and their obtained CT images were analyzed. The average age was 64.0 years ± 12.1 with an average BMI of 26.2 kg/m^2^ ± 5.4. According to the current WHO classification for BMI, the patient group can be described as being of a normal weight or slightly overweight. The STOP BANG risk questionnaire showed that two patients had a high risk (score ≥ 5), eleven patients had a moderate risk (score 3–4), and 37 patients had a low risk (score ≤ 2) of suffering OSA [3]. Most of the patients had oral squamous cell carcinoma (OSCC) in the region of the alveolar crest (mandibula) (24%), followed by OSCC of the tongue (20%) and OSCC of the palate (16%). Most patients were reconstructed with microvascular free flaps including the radial forearm transplants (48%) or local rotation flap (20%). Seventy-four percent of the patients received no radiation therapy (RTx), whereas 22% received only RTx and 4% received radiation therapy combined with chemotherapy (RCTx). In sum we generated 48 DICOM files for P_0_, 45 DICOM files for P_4_, and 37 DICOM files for P_8_.

### Minimum cross-sectional area

There was an increase in minCSA between the different protrusion steps (means: P_0_ = 236.4 mm^2^ ± 192.2; P_4_ = 309.2 mm^2^ ± 235.4; and P_8_ = 430.6 mm^2^ ± 265.3). Using a linear mixed model for repeated measures shows high significance (*p* < 0.001) for different protrusion steps. Calculating the increase of minCSA per millimeter by fixed effects estimated with a linear mixed model results in an enlargement of 24.9 mm^2^ per millimeter protrusion with a standard deviation (SD) of 13.0 mm^2^ (*p* < 0.001) (Table 1).

**Table 1.**
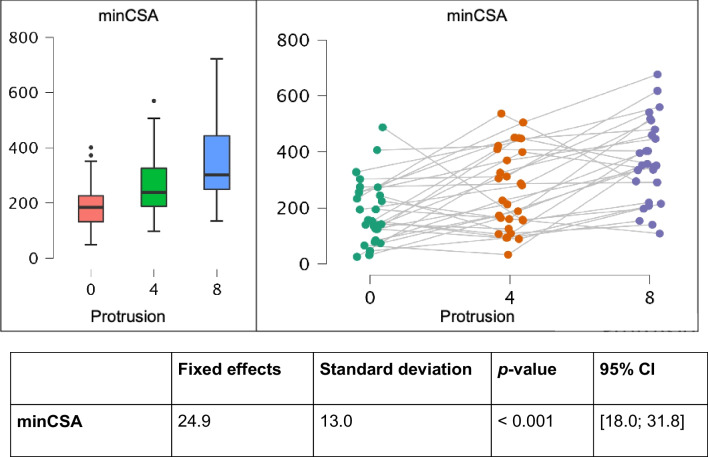
Minimum cross-sectional area (minCSA) linear mixed model analysis in mm^2^ and graphs

**Fig. 3:**
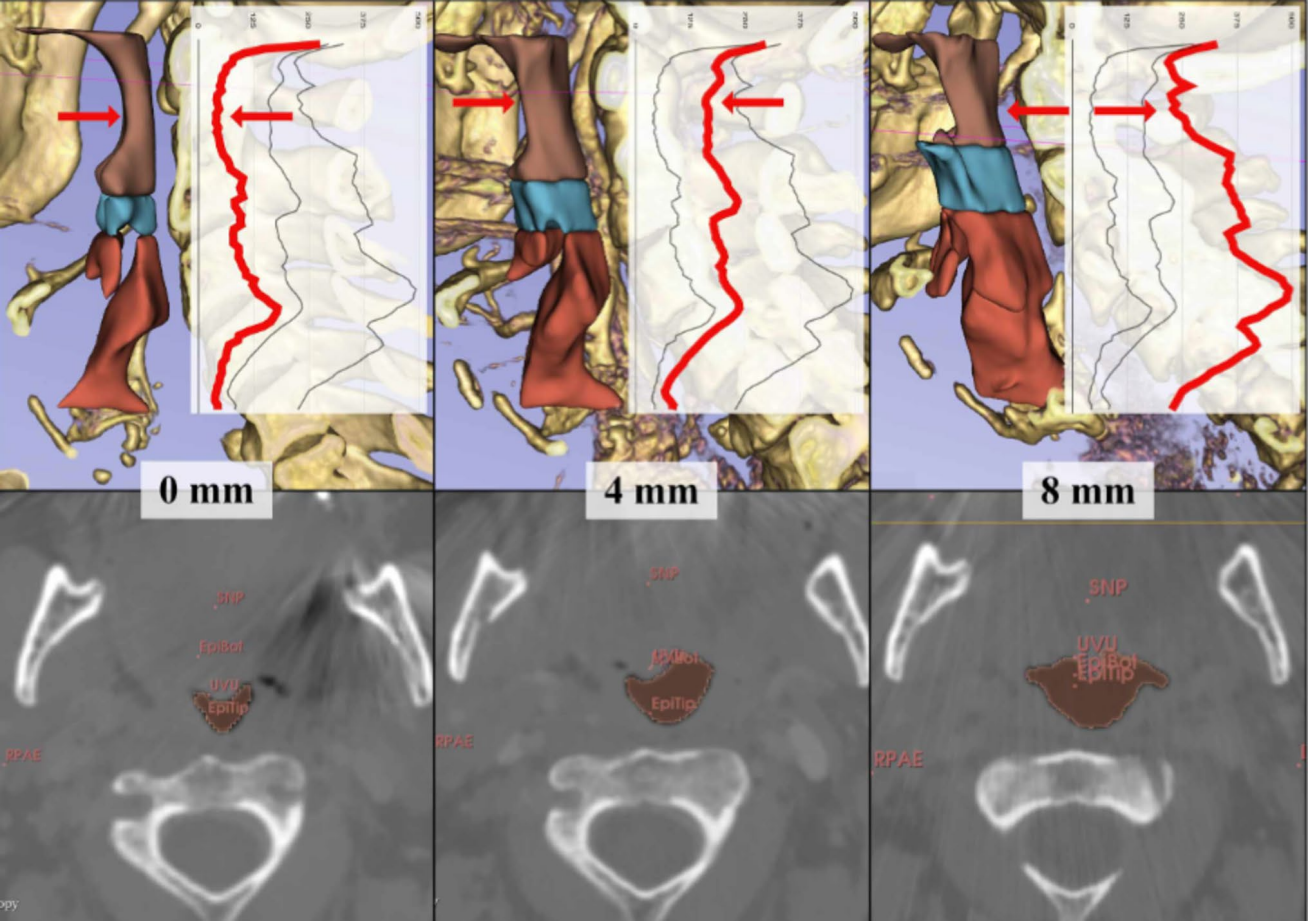
Illustration of the localization of the minimum cross-sectional area (minCSA) as a 3D rendering in sagittal view (A) and in CT in axial view

**Fig. 4:**
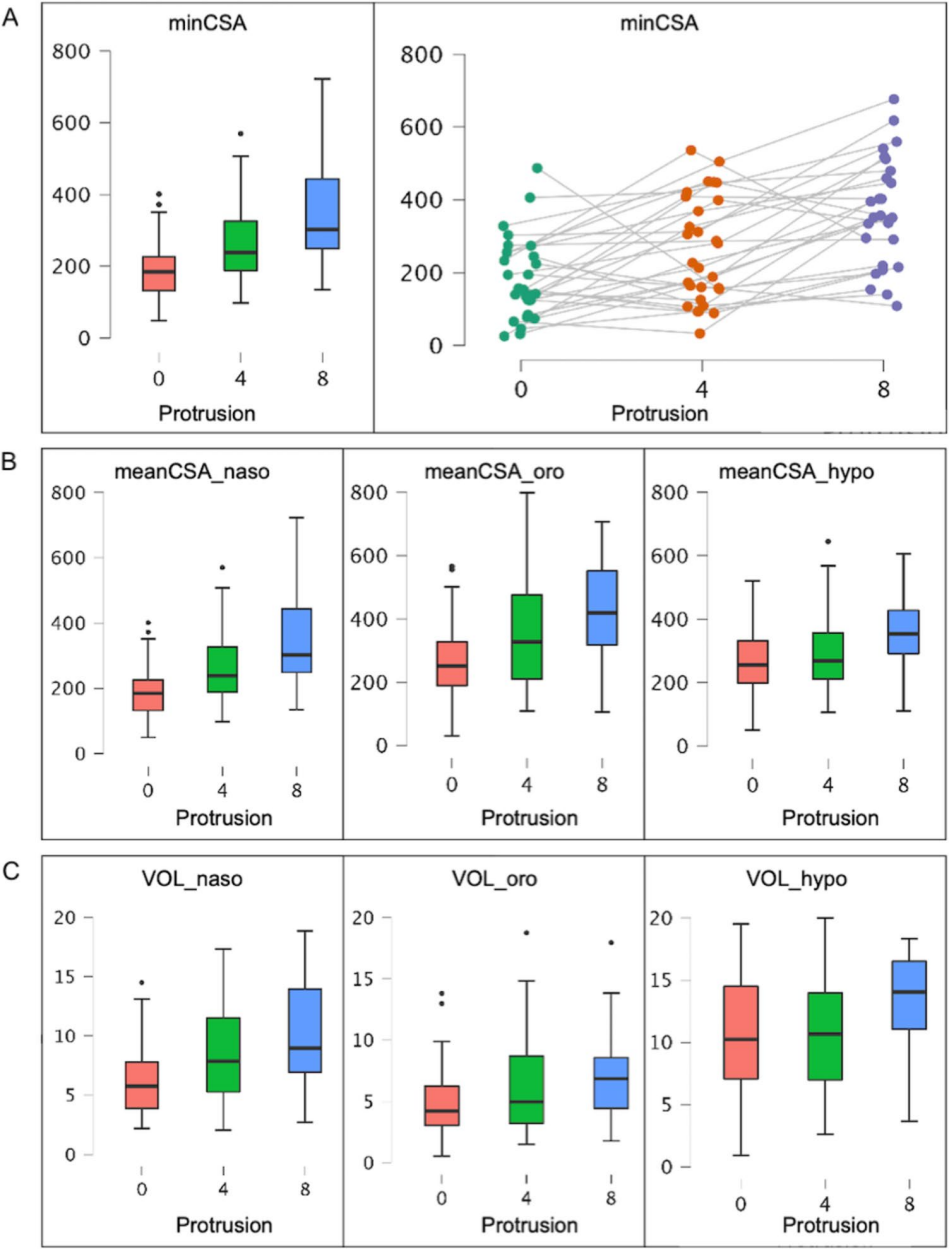
Illustration of linear mixed model analysis of (**A**) minimum cross-sectional area (minCSA), (**B**) mean cross-sectional area (meanCSA), and (**C**) volume in mm^2^ per millimeter protrusion

Without a MAD, 8.3% of the patients had a minCSA < 52 mm^2^, but with a MAD of 4 mm protrusion, only 2.2% had a minCSA < 52 mm^2^ and with a MAD of 8 mm protrusion none of the patients had a minCSA < 52 mm^2^. MinCSA is usually located in the nasopharynx, even in protrusion (minCSA located in the nasopharynx P_0_ = 91.7%, P_4_ = 86.7%, and P_8_ = 94.6%) (Fig. 3** and **Fig. 4** A**).

### Mean cross-sectional area

There is a highly significant (*p* < 0.001) increase in meanCSA in all protrusion steps and all parts of the pharynx of 20.0 mm^2^ (± 9.8) per millimeter. Calculating the increase of meanCSA per millimeter by fixed effects estimated with a linear mixed model results in an enlargement of 21.9 mm^2^ (± 11.2), 25.8 mm^2^ (± 14.1), and 13.9 mm^2^ (± 9.4) per millimeter protrusion (Fig. 4** B**).

### Volume

A significant enlargement of total volume with increasing mandibular advancement was registered (means: P_0_ = 24.0 cm^3^ ± 5.0; P_4_ = 29.6 cm^3^ ± 18.1; P_8_ = 33.6 cm^3^ ± 19.0) as well as for the section naso-, oro-, and hypopharynx. Linear mixed model analysis showed high significance for repeated measures (*p* < 0.001). Calculating the increase of volume per millimeter resulted in an increase of 1.61 cm^3^ per millimeter protrusion with a standard deviation of 0.70 cm^3^ (*p* < 0.001) (Fig. 4** C**).

### Impact of former OSCC localization, reconstruction, and radiation therapy on the PAS

No significant differences in any PAS parameters were seen by dividing the patient group into one group with mandibular bone involvement (n = 12) and another group containing all other OSCC localizations. Comparing the radial forearm flap (n = 24) with other reconstruction methods (n = 26) shows significantly higher airway parameters at P_4_ (*p* < 0.05) (Table 2). Patients who underwent radiation therapy (RTx or RCTx, n = 13) showed significantly (*p* < 0.05) higher airway dimensions than other patients (n = 37) (Table 2).

**Table 2:** Impact of reconstruction type (radial forearm flap versus other) and radiation therapy on the PAS parameter changes between the three different mandibular positions with reference (0 mm), 4 mm, and 8 mm protrusion

Parameter	Radial forearm flap	Other reconstruction	*p*-value
MinCSA – P_0_	275.3 ± 250.5	203.4 ± 119.6	0.200
MinCSA – P_4_	384.1 ± 286.2	243.7 ± 158.8	< 0.05
MinCSA – P_8_	503.7 ± 348.6	374.9 ± 167.2	0.146
MeanCSA – P_0_	282.9 ± 181.8	237.5 ± 83.8	0.260
MeanCSA – P_4_	383.4 ± 194.0	271.4 ± 98.4	< 0.05
MeanCSA – P_8_	429.9 ± 233.4	381.7 ± 124.8	0.424
Volume – P_0_	26.9 ± 20.0	21.6 ± 8.7	0.230
Volume – P_4_	36.6 ± 22.4	23.5 ± 10.4	< 0.05
Volume – P_8_	39.8 ± 25.9	32.3 ± 10.9	0.240
Parameter	RTx or RCTx	No radiation therapy	*p*-value
MinCSA – P_0_	334.2 ± 308.1	200.0 ± 112.0	< 0.05
MinCSA – P_4_	512.0 ± 361.8	243.6 ± 126.8	< 0.001
MinCSA – P_8_	579.5 ± 432.3	382.7 ± 169.4	0.051
MeanCSA – P_0_	318.5 ± 219.7	236.0 ± 85.8	0.065
MeanCSA – P_4_	440.6 ± 244.4	285.9 ± 98.4	< 0.01
MeanCSA – P_8_	488.7 ± 287.5	374.8 ± 121.8	0.097
Volume – P_0_	28.5 ± 24.3	22.4 ± 9.7	0.213
Volume – P_4_	39.6 ± 28.3	26.3 ± 12.3	< 0.05
Volume – P_8_	43.3 ± 32.2	33.1 ± 12.0	0.163

### Intra-class correlation

Fifteen patients were randomly selected, and software analysis was undertaken twice by the same operator (JZ). Statistical analyses of the measurements showed an ICC of 0.990 ± 0.02.

## Discussion

**Fig. 5:**
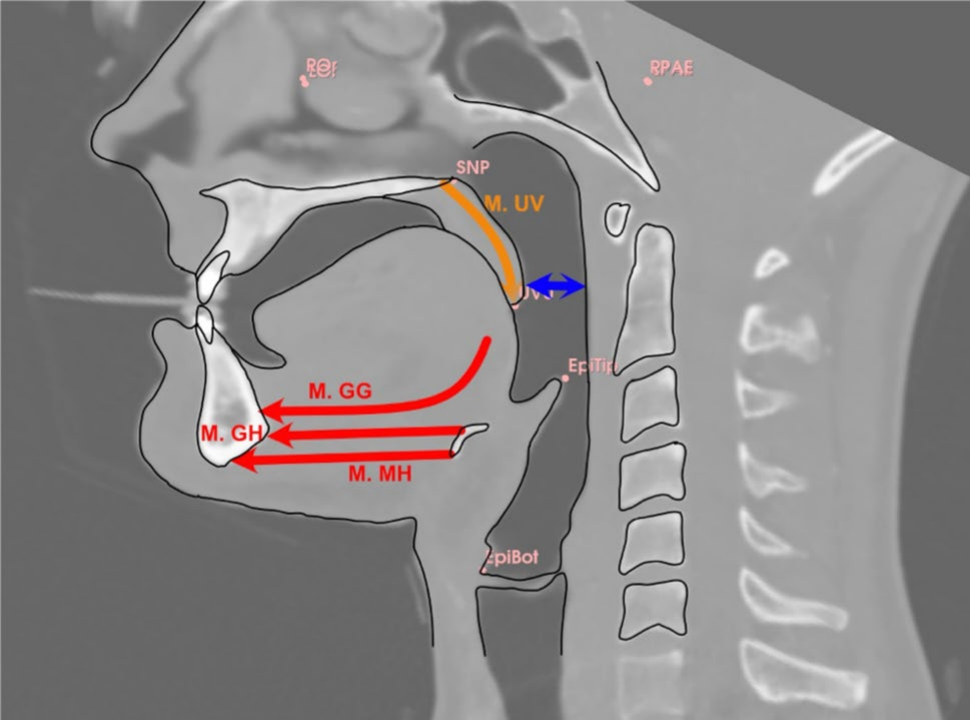
A simplified overview of the bony and muscular effects on the PAS that can result from MAD-derived mandibular protrusion: M. genioglossus (M. GG), M. geniohyoideus (M. GH), M. mylohydoideus (M. MH), M. palatoglossus (M. PG), M. uvulae (M. UV), M. constrictor pharyngis (M. CP)

The aim of this study was to get a better understanding of the interaction between temporary mandibular protrusion and PAS parameters. Additionally, this study aimed to describe quantitatively the correlation between the mandibular advancement distance and the increase in PAS parameters. The results showed a significant increase in minCSA, meanCSA, and volume with the amount of protrusion. The mean minCSA values in this study were consistent with those reported in similar research [34], though notable variability exists across studies, likely due to variations in reference points [38–40]. Additionally, meanCSA increased significantly across all airway sections (p < 0.001). As in Doff et al., the naso- and oropharynx showed relatively greater enlargement in our study compared to the hypopharynx [41]. However, Kochel et al. found the largest cross-sectional increase in the soft palate region [39]. Total volume and individual volume measurements in this study were similar to the absolute values reported by studies using the same reference points [31]. Combining the results of this study with Schendel et al. offers the possibility for calculating the optimal protrusion distance: one millimeter of protrusion resulted in an increase of 24.9 mm^2^ of minCSA. Investigating the preoperative minCSA of a patient shows how much increase of minCSA is needed to get a cross-sectional area of at least 110 mm^2^, thereby lowering the risk of suffering OSA [36]. In this context, the Hagen–Poiseuille equation claims that flow resistance is inversely proportional to the fourth power of cylindric radius and directly proportional to cylindric length, if one were to compare posterior airway space with a cylindric container. However, this law can be used to estimate airflow resistance in human airways [34]. Accordingly, minCSA is a very important parameter for airflow, and enlargement can effectively improve pharyngeal airflow. Accordingly, Giralt-Hernando et al. described that a mandibular advancement of 1 mm effected a 0.5 mm^3^ gain in the PAS and that for each 1 mm^3^ of PAS gain there was a decrease in AHI of 3.58 events/hour in two-dimensional measurements, which proved to be statistically significant [19]. Therefore the authors concluded that a greater change in the PAS would result in a lower final AHI. In our study, an almost doubling of minCSA resulted in a threefold airflow rate. Mandibular advancement seems to pull forward the hyoid bone via a pull effect of geniohyoid and mylohyoid muscles. The base of the tongue is also moved forward by the genioglossal muscle. Furthermore, the soft palate is placed in position against the base of the tongue, so the tip of the soft palate is also in a more ventral position (Fig. 4). Usually, minCSA was located at the level of the tip of the soft palate. Due to the ventral movement of soft tissue, minCSA slowly increases with increasing protrusion distance, as shown in Fig. 5. This interaction also makes it even more reasonable that the use of a MAD would also require lower pressures as part of nCPAP therapy, which could improve compliance with the conservative gold standard [11].

This anatomical interaction is also used for upper airway stimulation and leads to a constriction of genioglossal muscle and also an opening of the soft palate due to a palato-glossal muscle linkage [12]. In the past years, upper airway stimulation became a good therapy option for the treatment of moderate to severe OSA in patients with a BMI up to 35 kg/m^2^ [42]. However, the correlation of mandibular advancement and the increase in cross-sectional area and volume isn’t well understood yet [31]. Recent studies used two-dimensional imaging (lateral radiograph or cephalometric analysis) for the evaluation of posterior airway diameters where volume and cross-sectional area can’t be evaluated accurately [43–45]. Clinical limitations occur when imaging is taken in upright position and not in supine position. In supine position the distance between the tip of the palate and the posterior pharyngeal wall is significantly (*p* < 0.001) narrower than in upright position [46]. Our study is one of the few studies analyzing the PAS in supine position. This was possible because only oncologic aftercare CT scans were analyzed to avoid unnecessary radiation exposure according to the ethics and ALARA (as low as reasonably achievable) principles. This partly heterogeneous study population harbors the risk of selection bias, but a steady increase in the PAS parameters with an increase in the mandibular protrusion distance was nevertheless recorded. It remains to be seen whether this type of correlated increase in PAS parameters applies to patients who have not undergone enoral preoperative treatment radiotherapy. The minimum interval of six months after surgery and analyzed imaging was an attempt to minimize the misrepresentation of MAD effects due to general tissue reactions (decrease in graft volume and decrease in swelling) [21–24]. Other studies investigating the amount of mandibular advancement did not use absolute protrusion distance but described it as a proportion of maximum mandibular advancement. Using absolute distances for protrusion enables better comparison between different studies and is independent of limitations in patients’ maximum protrusion. Nevertheless, the interdisciplinary role of the orthodontist/dental specialist is secondary to the other sleep-medicine specialists and must be related to a preliminary assessment of MAD usage, testing a diagnostic MAD usable during a sleep examination (polysomnography or drug-induced sleep endoscopy) and final treatment with a definitive MAD, as stated by Lo Giudice et al. [47].

Getting the same cranio-cervical head position in each radiographic examination is difficult and it wasn’t possible to fully ensure this in this study. However, it seems to be that there is an impact of head tilt, hyoid position, and PAS. Additionally, most orthognathic-surgery-related studies of the PAS used CBCT in an upright position (standing or sitting). But since most problems occur when lying supine (supine-dominant OSA), this form of presentation does not adequately reflect reality. In this context, Ayoub et al. compared congruent CT and CBCT data from 55 patients and described that the CBCT significantly overestimates the PAS and depicts cross-sectional areas larger [48]. Thus, the position of the patient (upright versus supine) has a clear influence on the decisive parameters, which makes our findings relevant. Further studies are necessary in order to get a better understanding of this correlation. Segmentation is a very important part of airway analysis and can be done manually or automatically. Manual segmentation is very time-consuming because boundaries must be set slice by slice and then rendered to a 3D model. Using an automatic segmentation algorithm is more convenient but also generates more errors [49]. Combining manual segmentation with automatic functions provides fast segmentation while minimizing errors. In this study we used the “grow from seeds” function of 3D Slicer, where different tissue and airway areas were marked by “seeds” and then computed together to create a 3D model. If errors occur while using this growing algorithm, they can be manually corrected.

### Limitations

In this study the STOP BANG questionnaire was used. This is a validated and fast screening questionnaire to determine the risk of sleep apnea by assessing the signs, symptoms, and risk factors of OSA, including snoring, drowsiness, witnessed apnea, obesity, and hypertension. The Epworth Sleepiness Scale (ESS) would have been an alternative, one which is often used in the literature due to its simplicity [26]. However, it also has a lower diagnostic value because it measures the general level of sleepiness. Other questionnaires like SF-36 or Functional Outcomes of Sleep (FOSQ) need to be performed twice, which was not in concordance with our study design. The study population consisted of patients who were treated and followed up because of OSCC. This might result in bias due to study population selection because radiation-induced edema and fibrosis can narrow the airways, reducing their volume and potentially causing compromising airway patency over time, which results in characteristic imaging changes in the head and neck region [50]. On the other hand there is also evidence in the literature that radiation may induce higher volume losses in free microvascular flaps – especially in antero-lateral thigh flaps – over time [21]. However, even in this study population, a temporarily applied MAD had a significant effect on the analyzed PAS parameters. Only in this special study population was it ethically and for radiation protection reasons possible to obtain three consecutive CT scans with different mandibular positions in supine position. This in turn distinguishes this study despite the selection bias.

## Conclusion

The results of this study may serve surgeons and dentists to predict the change in posterior airway space dimensions more accurately. This information could result in better planning in orthognathic surgery and conservative and surgical OSAS treatment.

## Data Availability

No datasets were generated or analysed during the current study.

## Abbreviations

AHI: Apnoea–hypopnoea index
BMI: Body mass index
CBCT: Cone-beam computed tomography
CT: Computed tomography
EpiBot: Bottom of the epiglottis
EpiTip: Tip of the epiglottis
ICC: Intra-class correlation coefficient
LOr / ROr: Left / right orbitale
LPAE / RPAE: Left / right porus acusticus externus
MAD: Mandibular advancement devices
meanCSA: Mean cross-sectional area
minCSA: Minimum cross-sectional area
nCPAP: Nasal continuous positive airway pressure
OSA: Obstructive sleep apnea
OSCC: Oral squamous cell carcinoma
P_0_: 0 Mm protrusion
P_4_: 4 Mm protrusion
P_8_: 8 Mm protrusion
PAS: Posterior airway space
RCTx: Radiation therapy combined with chemotherapy
RTx: Radiation therapy
SD: Standard deviation
SNP: Posterior nasal spine
UVU: Tip of the uvula
VOL: Volume

## Declarations

### Clinical trial

This manuscript does not report on a clinical trial.
